# Effectiveness of Erbium: YAG Laser in the Treatment of Vulvovaginal Atrophy in Women Who Survived Breast Cancer

**DOI:** 10.1111/jocd.70424

**Published:** 2025-09-12

**Authors:** Keila Seabra Teles Ferreira, Giovanna Andrade Lopes, Letícia Alves Barbosa, Israel Júnior Borges do Nascimento, Moisés Salgado Pedrosa, George Kroumpouzos, Gisele Viana de Oliveira

**Affiliations:** ^1^ Department of Post‐Graduation Faculty of Medical Sciences of Minas Gerais Belo Horizonte Brazil; ^2^ Division of Country Health Policies and Systems (CPS) World Health Organization Regional Office for Europe Copenhagen Denmark; ^3^ Department of Internal Medicine at the School of Medicine and Hospital Universitário Clementino Fraga Filho Federal University of Rio de Janeiro Rio de Janeiro Brazil; ^4^ Department of Pathology Medical College of Wisconsin Milwaukee Wisconsin USA; ^5^ Department of Pathology Clinical Hospital of the Federal University of Minas Gerais Belo Horizonte Brazil; ^6^ Department of Dermatology Warren Alpert Medical School at Brown University Providence Rhode Island USA; ^7^ GK Dermatology, PC South Weymouth Massachusetts USA; ^8^ Dermatologic Surgery Clinic Santa Casa de Misericórdia de Belo Horizonte Belo Horizonte Minas Gerais Brazil

**Keywords:** atrophic vaginitis, breast cancer, cancer survivors, erbium, laser therapy

## Abstract

**Background:**

Genitourinary syndrome of menopause (GSM) is prevalent among breast cancer survivors (BCS), often exacerbated by oncologic treatments and compounded by contraindications to hormone‐based therapies. Vaginal Erbium:YAG laser has emerged as a promising non‐hormonal alternative, though long‐term safety and efficacy data remain scarce.

**Methods:**

This prospective pilot study initially enrolled twelve breast cancer survivors (BCS) presenting with moderate to severe genitourinary syndrome of menopause (GSM). The study consisted of two phases: in phase 1, patients underwent 3 monthly sessions of fractional Erbium:YAG (erbium: yttrium‐aluminum garnet) laser and were evaluated before the first laser and 1 month after the third session. In Phase 2, patients were evaluated 1 year after the beginning of the treatment (first laser session). Clinical evaluations were carried out at baseline and after each study phase using the Vaginal Health Index (VHI), the Female Sexual Function Index (FSFI), and a customized Symptom Intensity Questionnaire. Vaginal biopsies and cytological samples were obtained at baseline and at the end of the first phase. H&E staining, Picrosirius Red, glycogen deposits, and epithelial thickness were evaluated in all samples. A Likert scale was administered at the end of each phase to assess patient satisfaction. Statistical analyses were performed using paired *t*‐tests, bootstrap resampling techniques, and logistic regression models to evaluate treatment outcomes and response predictors.

**Results:**

12 patients enrolled in the study, ten completed phase one, and eight completed Phase 2. Mean VHI scores improved significantly from 10.75 ± 2.4 to 23.38 ± 3.1 (*p* < 0.0001). Symptom severity decreased significantly (*p* = 0.0078). FSFI scores showed clinical improvement (from 15.52 ± 7.20 to 25.05 ± 6.81), though not statistically significant (*p* = 0.0889); bootstrap and simulation analyses confirmed robustness. Histological findings indicated epithelial remodeling without adverse tissue effects. All patients gave the best satisfaction scores to the treatment (Likert = 5). No adverse events were reported.

**Conclusions:**

Vaginal Erbium:YAG laser therapy appears to be a safe and potentially effective option for GSM management in BCS. Further randomized controlled trials are needed to validate these findings.

## 1 Introduction

Breast cancer is the most frequently diagnosed cancer in women worldwide [1]. The treatment of breast cancer is multidisciplinary; it includes locoregional therapeutic approaches (surgery and radiotherapy) as well as systemic therapies [2]. Systemic therapies comprise endocrine therapy for hormone receptor–positive disease, chemotherapy, anti‐HER2 therapy for HER2‐positive disease, bone‐stabilizing agents, poly (ADP‐ribose) polymerase inhibitors for BRCA mutation carriers, and, more recently, immunotherapy [2]. Approximately 42% to 70% of patients receiving systemic treatment for breast cancer, including endocrine therapy, are likely to experience some degree of vulvovaginal atrophy (VVA) [3]. Chemotherapy can cause sudden, temporary, or permanent menopause in up to 80% of premenopausal women due to premature ovarian failure, in addition to potentially leading to a further reduction in estrogen levels after natural menopause [4].

Menopausal Genitourinary Syndrome (MGS) encompasses a variety of symptoms resulting from decreased estrogen levels: vaginal dryness, irritation, and dyspareunia, as well as urinary manifestations such as dysuria, urgency, and recurrent urinary tract infections [5, 6]. In breast cancer survivors (BCS), MGS has been identified as the primary factor limiting post‐cancer quality of life, underscoring the discomfort of its symptoms and the importance of treating them [7]. These symptoms may be more severe due to the adverse effects of oncological treatments, such as chemotherapy and endocrine therapies, which induce iatrogenic menopause or intensify the hypoestrogenic state [8]. The initial management of MGS includes the use of vaginal moisturizers and lubricants, but these approaches often provide insufficient symptom relief [9]. Furthermore, although local hormonal therapies are effective, they are generally used with caution in patients with a history of breast cancer due to the potential risk of tumor recurrence [10].

In recent years, vaginal laser therapy with Erbium:YAG (erbium: yttrium‐aluminum garnet) 2940 nm and CO_2_ laser 10 600nm has emerged as a possible adjuvant treatment for SGM, especially when patients are not ideal candidates for hormonal therapies [11, 12]. Among the available modalities, the erbium:YAG laser has stood out for its nonablative photothermal effects, which promote the remodeling of vaginal tissue and neocollagenesis, resulting in improved lubrication and elasticity of the vagina, urethra, bladder, or rectum [13]. Preliminary studies indicate that treatment with the erbium:YAG laser can significantly alleviate the symptoms of MGS in breast cancer survivors, with a favorable safety profile and few reported adverse events [14]. The laser parameters are set to achieve a controlled profound thermal effect without tissue ablation or carbonization, thereby avoiding the risk of perforation from accidental injury to the vaginal mucosa, urethra, bladder, or rectum [15]. However, despite these promising results, scientific evidence regarding the long‐term efficacy and safety of the erbium:YAG laser for the treatment of MGS in breast cancer survivors remains limited [16]. Most available studies are observational, with small sample sizes and short‐term follow‐ups. This underscores the need for further research, including randomized clinical trials with extended follow‐up to validate these findings and establish standardized therapeutic protocols [17]. Healthcare professionals must remain vigilant to the symptoms of MGS and offer individualized management, considering each patient's preferences and contraindications, to improve their quality of life [7].

In this context, this study aims to comprehensively evaluate the efficacy and safety of the erbium:YAG laser in the treatment of Menopausal Genitourinary Syndrome in breast cancer survivors, providing data for physicians and other healthcare professionals who work directly with this specific population and contributing to the development of evidence‐based therapeutic strategies.

We structured this study to answer two questions: “Is the erbium:YAG laser a safe treatment for breast cancer survivors?” and “Is laser treatment effective for managing genitourinary syndrome in patients with breast cancer?”

## 2 Materials and Methods

### 2.1 Study Design and Ethical Aspects

This prospective study was approved by the Research Ethics Committee of the Faculty of Medical Sciences of Minas Gerais—FCMMG under number CAAE 75613723.5.0000.5134. All participants were duly informed about the procedures and objectives of the study through the Free and Informed Consent Form. The study was conducted between September 2023 and January 2025. Since there are still few studies on the subject, there was insufficient data for a sample size calculation. The sample included 12 breast cancer survivors, recruited from the Mastology Health Care System in the city of Varginha, MG. For inclusion in the study, patients had to meet the following criteria: age between 30 and 70 years, a history of hormone‐dependent breast cancer and use of hormone blockers, completion of chemotherapy treatment, complaint of at least three symptoms of genitourinary syndrome of menopause, and agreement with the informed consent form for participation in the study. The exclusion criteria were: patients who did not wish to have their data used in the study, those who refused to undergo biopsy and cytological examination before or after the treatment, pregnant and lactating patients, and finally patients who had previously undergone vaginal laser treatment.

**FIGURE 1:**
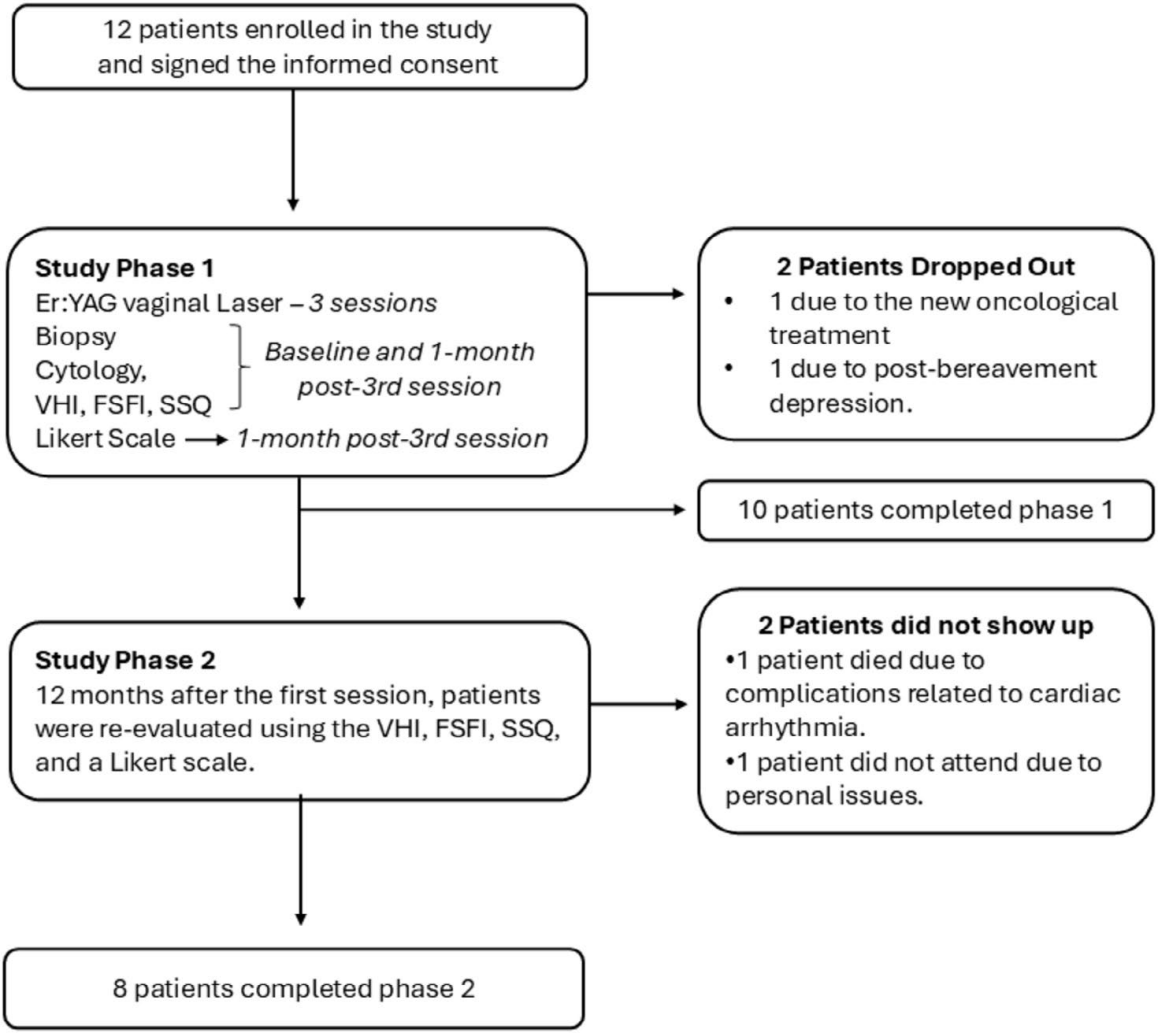
Study CONSORT diagram of the prospective clinical study evaluating Er:YAG laser therapy in BCS with GSM: The diagram depicts the progression of participants throughout the study, including recruitment, intervention, follow‐up assessments, and reasons for discontinuation. Ten patients completed the first phase, which included three sessions of non‐ablative vaginal Er:YAG laser therapy and post‐treatment evaluation, while eight patients completed the final assessment twelve months after treatment initiation. Outcomes were assessed through biopsy, cytology, the Vaginal Health Index (VHI), the Female Sexual Function Index (FSFI), a symptom severity questionnaire (SSQ), and a Likert scale. BCS, breast cancer survivors; GSM, genitourinary syndrome of menopause.

The study consisted of two phases. Phase 1 included three laser treatment sessions and evaluations immediately before the first session and one month after the third session. Phase 2 consisted of a new assessment one year after the baseline, the beginning of the study. Twelve patients enrolled in the study, and ten patients completed Phase 1. One patient dropped out of the study due to the diagnosis of metastasis and the new oncological treatment. Another patient abandoned the study due to post‐bereavement depression. Eight patients completed Phase 2. In Phase 2, one patient did not attend the appointment due to personal reasons, and another patient died nine months after the end of Phase 1, due to complications of cardiac arrhythmia. Therefore, eight patients were evaluated one year after the initial treatment (Figure 1).

### 2.2 Study Evaluation Tools

#### 2.2.1 Female Sexual Function Index (FSFI) Questionnaire

On the day of the first session, the patients completed the Female Sexual Function Index (FSFI) questionnaire, consisting of 19 questions covering six domains of female sexual function: 1. sexual desire, 2. sexual arousal, 3. vaginal lubrication, 4. orgasm, 5. sexual satisfaction, and 6. sexual pain (dyspareunia and vaginal discomfort) [18]. For each domain, there are a few questions that receive different grades. Each question is scored from 0 to 5, with a total FSFI score ranging from 2 to 36 (Table 1). The interpretation of the results considers the following values: an FSFI score equal to or greater than 26.55 indicates normal sexual function. In contrast, a score below 26.55 suggests the presence of female sexual dysfunction [19].

**TABLE 1:** Calculation parameters for the Female Sexual Function Index (FSFI).

Domain	Items	Response range	Domains factor	Minimum score	Maximum score
Desire	1–2	1–5	0.6	1.2	6.0
Arousal	3–6	0–5	0.3	0	6.0
Vaginal lubrication	7–10	0–5	0.3	0	6.0
Orgasm	11–13	0–5	0.4	0	6.0
Satisfaction	14–16	0/1–5	0.4	0.8	6.0
Sexual pain	17–19	0–5	0.4	0	6.0

*Note:* Scoring structure of the Female Sexual Function Index (FSFI), including domain‐specific items, response ranges, and weighting factors used to calculate the final score.

#### 2.2.2 Vaginal Health Index (VHI)

During the physical examination, the Vaginal Health Index (VHI) was applied, which consists of five clinical parameters evaluated during the gynecological exam: elasticity (the ability of the vaginal mucosa to stretch without causing pain or fissures), vaginal discharge (amount of lubrication present), vaginal pH (usually below 4.5 in reproductive‐age women and above 5.0 in cases of vaginal atrophy), vaginal epithelium (assessment of mucosal thickness and integrity), and vaginal moisture (degree of mucosal hydration). Each parameter is scored from 1 to 5, where 1 corresponds to severe atrophy, 2 to 3 indicates moderate atrophy, and 4 to 5 corresponds to a normal mucosa. The total VHI score ranges from 5 to 25 points [20]. For interpretation, values equal to or greater than 15 indicate a healthy vaginal mucosa with no significant signs of atrophy; values ranging from 11 to 14 indicate mild to moderate atrophy with subtle symptoms; and values equal to or less than 10 indicate severe vaginal atrophy, with significant symptoms and a need for intervention [20, 21]. Vaginal pH, one of the parameters of the VHI, was measured using pH indicator strips (range 4.0–7.0; MColorpHast; Merck, Germany) applied against the vaginal wall, with the reading performed according to the test instructions.

#### 2.2.3 Symptom Intensity Questionnaire

In addition to assessing the intensity of symptoms, this questionnaire also evaluated the impact of these symptoms on the patients' quality of life. Each patient answered the questionnaire before the first session and at the end of each phase. The questionnaire was developed using artificial intelligence and based on GSM symptoms reported in the literature [22, 23, 24]. This instrument assesses the following symptoms: vaginal dryness, vaginal burning or irritation, vaginal itching, dyspareunia, decreased lubrication, bleeding and micro fissures after sexual intercourse, urinary urgency, polyuria, recurrent urinary tract infections, sensation of incomplete bladder emptying, stress urinary incontinence, and dysuria. Regarding the impact on quality of life, the questionnaire covers the effects on sexual life, emotional well‐being, and daily activities. The score ranges from 0 to 48; for interpretation, values from 0 to 10 indicate mild symptoms with little impact on quality of life, 11 to 24 indicate moderate symptoms that may require clinical intervention, and 25 to 48 indicate severe symptoms that justify therapeutic intervention.

#### 2.2.4 Likert Scale

At the end of the first phase, patients completed the Likert scale regarding symptom improvement. At the end of the second phase, the Likert scale was applied regarding the maintenance of improvement after one year.

#### 2.2.5 Cytological Examination

Cytological samples were obtained from the vaginal wall using a cytology brush, which was subsequently placed into the liquid medium vial provided in the collection BD Sure Path kit. Sampling was conducted before the first treatment session, before the procedure. Cytology was used to investigate cellular atypia.

#### 2.2.6 Biopsy

Immediately before the first session, biopsy specimens were collected for histopathological analysis. A 3 mm punch biopsy was performed in the inferior region of the vaginal introitus, with the patient positioned in gynecological lithotomy. The procedure was carried out under topical local anesthesia, with the addition of an anesthetic bleb, and the site was sutured using Vicryl 6.0. Tissue sections were processed using the paraffin‐embedding technique. A second biopsy specimen was collected one month after the third laser treatment.

#### 2.2.7 Epithelial Thickness

Epithelial thickness was measured with a micrometric eyepiece, from the basal layer to the surface of the epithelium, considering both the thinnest and thickest areas. The analysis was based on each sample's smallest and largest measurements.

#### 2.2.8 Glycogen Deposits

The glycogen deposits were evaluated using periodic acid‐Schiff (PAS) staining, a histochemical technique highlighting polysaccharide components within tissues. The assessment was conducted under light microscopy, with a pathologist performing a semi‐quantitative evaluation based on staining intensity and distribution. Glycogen content was categorized as follows: sparse (+), slight (++), moderate (+++), and pronounced (++++).

#### 2.2.9 Picrosirius Staining for Collagen

Special staining with Picrosirius Red (Sirius Red) was employed to visualize collagen fibers. Microscopic images were captured via optical microscopy and subsequently analyzed using custom software developed in Python. Specific HSV (hue‐saturation‐value) ranges were defined to segment collagen‐associated color bands—pinkish‐red, burgundy, and light purple. The proportion of collagen was determined by comparing the number of segmented pixels within the defined stain spectrum to the total pixel count (including unstained and irrelevant colored pixels) in the dermal region, thereby quantifying collagen fiber density before and after treatment.

### 2.3 Therapeutic Intervention

After the study was approved in the Ethical Committee, patients signed the informed consent and explained about the study, where three vaginal laser sessions would be performed at a single Private Setting in Varginha, MG, Brazil. Immediately before the first laser session, they completed the FSFI and *Symptom Intensity Questionnaire*. Then, two of the investigators jointly examined with a speculum to calculate the Vaginal Health Index. After that evaluation, the vaginal introitus was anesthetized using a 7% lidocaine cream, later removed with saline solution. The vaginal laser used was a 2940‐nm Erbium:YAG laser (Etherea‐MX, Athena, São Carlos, São Paulo, Brazil), with 90° and 360° scanning probes. In the gynecological position, the 90° probe was inserted into the speculum after the device‐specific fenestrated speculum. The anterior vaginal wall was irradiated with three pulses every 10 mm (using the scale on the tip of the device). This procedure was repeated three times up to the entrance of the vaginal canal, with the speculum rotated to the 11, 12, and 1 o'clock positions. The parameters used for this collimated tip were: fluence (laser energy delivered per unit area) of 30 mJ/MTZ (equivalent to 10.37 J/cm^2^), frequency of 0.5 Hz, and smooth‐mode pulses. After the removal of the first scanning probe, the 360° scanning probe was inserted into the speculum, without direct contact with the vaginal mucosa. The vaginal wall was irradiated in 360° with three pulses every 5 mm (using the scale on the tip of the device). This procedure was repeated three times up to the entrance of the vaginal canal. The parameters used for this collimated tip were: fluence (laser energy delivered per unit area) of 2.5 J/cm^2^, frequency of 0.5 Hz, and smooth‐mode pulses. The InLift probe was used on the vaginal introitus, with an energy of 30 mJ/MTZ and 10 shots along the entire introitus. This process does not generate vaporization; it only heats the tissue and helps stimulate collagen production without causing necrosis [25].

All patients underwent three laser sessions at 30‐day intervals. One month after the last session, they completed a sexual function questionnaire (FSFI), the Likert scale, their Vaginal Health Indexes were reassessed, and new cytology and biopsy samples were collected. After one year, the patients returned; once again, they completed the sexual health questionnaire (FSFI), a Likert scale, and a symptom intensity questionnaire referring to the beginning of the study, the end of the first phase, and the current symptoms. During the speculum exam, the Vaginal Health Index was reassessed, and the patients received a maintenance session.

### 2.4 Data Analysis and Processing

We initially summarized continuous variables utilizing means and standard deviations (SDs) or medians and interquartile ranges (IQRs), based on the calculated distribution evaluated through the Shapiro–Wilk test. Categorical variables were reported as frequencies and percentages. To compare pre‐ and post‐treatment outcomes, we utilized the paired *t*‐test (among normally distributed variables) and the Wilcoxon signed‐rank test (for non‐normally distributed variables). As we enrolled a limited number of patients, we performed a bootstrap resampling with 1000 iterations for estimating confidence intervals (CIs) for the mean difference in the Female Sexual Function Index (FSFI). Moreover, we created a simulated dataset of an increased cohort (*n* = 20) to assess whether the limited sample size influenced the calculated statistical significance through normal distributions resulting from the primary data.

We also explored potential factors related to treatment response. For this, we categorized patients into two categories (Responders and Non‐Responders) according to their median improvement in FSFI scores. Whenever appropriate, the differences between responders and non‐responders were analyzed using independent *t*‐tests or Mann–Whitney *U* tests. Additionally, we analyzed different variables (i.e., age at diagnosis, baseline Vaginal Health Index—VHI, and initial symptoms severity predicted treatment response) using binary logistic regression modeling. Model appropriateness was evaluated using deviance residuals, with findings expressed as odds ratios (ORs) with associated 95% CI. All statistical analyses were two‐tailed and considered statistically significant with a *p*‐value < 0.05. We used R version 4.4.1 (R Foundation for Statistical Computing, Vienna, Austria) for obtaining all statistical assessments.

## 3 Results

### 3.1 Baseline Characteristics

Initially, 10 patients with breast cancer who had completed phase 1 were enrolled, with a mean age at diagnosis of 47.7 years (SD = 10.3). However, two patients were excluded from the analysis due to one death (unrelated to the intervention delivered) and one drop‐out. Therefore, eight patients (meaning age at diagnosis of 51.8 years [SD = 9.1]) were included in our final analysis. At baseline, sexual function (represented as FSFI), vaginal health (defined as VHI), and genitourinary symptom severity suggested significant impairment. At the first time point (beginning of the treatment), FSFI score was 15.6 (SD = 7.4), while VHI score was 10.75 (SD = 2.04), and the severity of genitourinary symptoms (examined using a pre‐validated structured questionnaire) yielded a median score of 26.5 (IQR: 19.5–33.3). Furthermore, at the end of the study, all patients categorized their satisfaction with the intervention as highly adequate (Likert for all patients = 5).

### 3.2 Sexual Function Outcomes (FSFI Measures)

**FIGURE 2:**
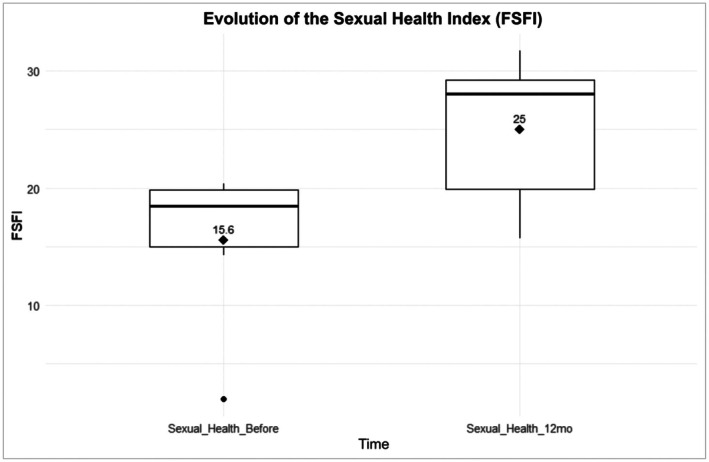
Progression of FSFI following vaginal laser therapy in BCS: Twelve months after the intervention, variables related to female sexual health were reassessed. The graph illustrates that the mean FSFI score increased from 74.5 (SD = 11.7) at baseline to 97.5 (SD = 12.6), indicating a notable improvement. Although the paired *t*‐test demonstrated a trend toward improvement in the FSFI score, the estimates generated did not reach the threshold for statistical significance (*p* = 0.0889). BCS, breast cancer survivors; FSFI, Female Sexual Function Index.

At twelve months post‐intervention, variables related to female sexual health were reassessed. The mean FSFI score showed a notable increase from baseline (15.5 [SD = 7.20]) to 25.0 (SD = 6.81), as illustrated in Figure 2. Although the paired *t*‐test indicated a trend toward improvement, the change did not reach conventional statistical significance (*p* = 0.08893). As described in the methodology, additional statistical analyses were performed to assess the robustness of the findings, given the small sample size. A bootstrap model with 1000 resampling iterations confirmed a clinically meaningful improvement in FSFI scores, yielding a 95% confidence interval ranging from −0.33 to 40.00. To explore whether the limited sample size had impacted the observed results, a simulated dataset of 20 patients was generated based on the structure of the primary data. This simulation revealed statistically significant variance in FSFI improvement (*p* = 0.0008), supporting the hypothesis that the original analysis may have lacked sufficient power to detect the full magnitude of the treatment effect.

Among the eight participants who completed the 12‐month follow‐up, two did not have a sexual partner; therefore, the FSFI analysis was conducted with six patients. Participants whose FSFI improvement exceeded the cohort median were classified as responders, whereas those with changes below the median were considered non‐responders. Baseline factors such as vaginal health (*p* = 0.1635), age at diagnosis (*p* = 0.2297), and symptom severity (*p* = 0.9222) were assessed for predictive value, but none demonstrated statistically significant associations with treatment response.

### 3.3 Vaginal Health Index (VHI Measures)

**FIGURE 3:**
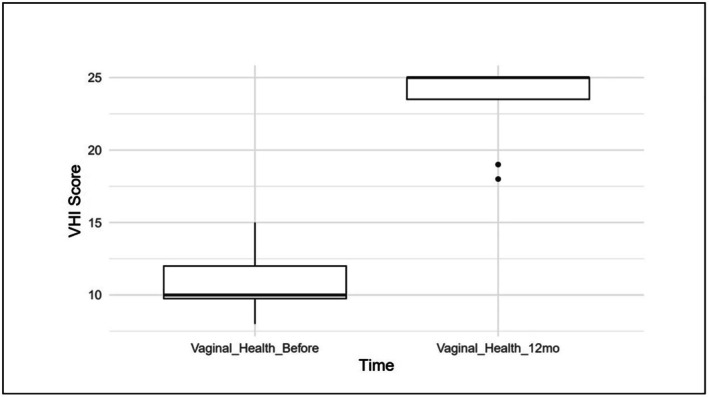
Progression of VHI following vaginal laser therapy in BCS: The graph compares VHI scores before the procedure and 12 months after the initiation of treatment. A statistically significant improvement was observed, as indicated by the paired *t*‐test (*p* < 0.0001). The mean VHI score at baseline was 10.75 (SD = 2.4), increasing to 23.38 (SD = 3.1) at the 12‐month follow‐up. BCS, breast cancer survivors; VHI, Vaginal Health Index.

Our analysis comparing the VHI at baseline and 12 months revealed a statistically significant improvement following our intervention (*p* < 0.0001) (Table 2). At baseline, the average VHI score was 10.75 (SD = 2.4), whereas in 12 months, the average VHI score was 23.38 (SD = 3.1) (Figure 3).

**TABLE 2:** Evolution of the parameters that make up the VHI after vaginal laser treatment in breast cancer survivors.

Symptoms	Before the intervention	After the intervention	One year after the intervention
Vaginal moisture	2.6	4.9	5.0
Volume of vaginal fluid	1.5	5	4.9
pH	2.4	3.7	4.1
Elasticity	1.8	4.8	4.8
Epithelial integrity	2.5	4.4	4.5

*Note:* Progression of mean scores for each parameter of the Vaginal Health Index (VHI) among breast cancer survivors (BCS). Assessments were conducted at three time points: before treatment, one month after treatment completion, and one year after treatment initiation. Each parameter is scored from 1 to 5, where 1 indicates the most severe condition and 5 represents normality. Improvements were observed across all parameters, with vaginal moisture reaching normal levels and other parameters approaching normal values.

Abbreviations: BCS, breast cancer survivors; VHI, Vaginal Health Index.

### 3.4 Symptom Intensity Questionnaire

**FIGURE 4:**
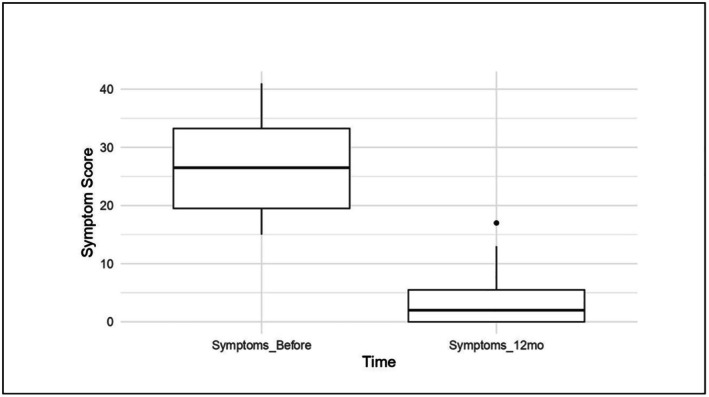
Progression of GSM symptom severity following vaginal laser therapy in BCS: The graph shows the evolution of GSM symptoms, with a significant and statistically significant reduction in the paired *t*‐test in complaints after treatment with Erbium:YAG laser. 22 points reduced the median severity of symptoms from baseline to 12‐month follow‐up (*p* = 0.0078).

The severity of genitourinary symptoms (estimated using the self‐administered questionnaire developed based on previous studies [21, 22, 23]) revealed a statistically significant reduction in complaints after the Erbium:YAG laser treatment (Table 2 and 3). Twenty‐two points reduced the median severity of symptoms from baseline to the 12‐month follow‐up (*p* = 0.0078) (Figure 4).

### 3.5 Lickert Scale

All patients rated the treatment with the maximum grades for satisfaction both after Study Phase 1 and Phase 2.

### 3.6 Comparison Between Responders and Non‐Responders and Predictors of Treatment Response

We performed complementary analyses to assess whether baseline characteristics influenced treatment‐related outcomes, categorizing individuals as responders (patients with a change in FSFI score > median FSFI score) and non‐responders (patients with a change in FSFI score < median FSFI score). Our comparisons between these two groups revealed no significant variation in the main baseline characteristics. It is worth noting that the mean age at diagnosis for patients classified as responders was 47.7 years, whereas for those classified as non‐responders, it was 59.7 years (*p* = 0.2297). Similarly, the baseline VHI was 12.33 in responders compared to 9.33 in non‐responders (*p* = 0.1635). Symptom severity at baseline was also comparable between responders and non‐responders (27.3 and 26.3, respectively), with no statistical significance (*p* = 0.9222). We also developed a logistic regression model to evaluate whether three variables could predict treatment response. However, age at diagnosis, baseline VHI, and baseline symptom severity showed no significant association.

### 3.7 Histological Evaluation

No statistically significant differences were observed in histological architecture before and after treatment. Although all patients exhibited Vaginal Health Index (VHI) scores consistent with moderate to severe Genitourinary Syndrome of Menopause (GSM), with a notable impact on quality of life at baseline, only one case demonstrated cytological evidence of atrophy. No cellular atypia was detected at any time during cytological assessment.

### 3.8 Vaginal Tissue Biopsies

Histological examination of vaginal tissue biopsies obtained before and after Erbium:YAG laser treatment revealed morphological changes potentially attributable to the intervention.

**TABLE 3:** Evolution of the intensity of each symptom after vaginal laser treatment in BCS.

Symptoms	Before the Erbium YAG laser	One month after the intervention	One year after the intervention
Vaginal dryness	2.5	0.25	0.125
Vaginal burning	2.25	0.375	0.125
Vaginal itching	0.625	0.125	0.25
Dyspareunia	1.85	0.714	0.428
Lack of lubrification	2.57	0.857	0.714
Bleeding/micro fissures after intercourse	0.714	0	0
Urinary urgency	1.75	0	0.25
Polyuria	1.875	0.625	0.25
Nocturia	1.5	0.75	0.5
Recurrent urinary tract infections	1.75	0.125	0.125
Incomplete bladder emptying	1.375	0.75	0.625
Stress urinary incontinence	1.5	0.375	0.125
Dysuria	0.375	0.125	0
Effects on emotional well‐being	2.5	0.625	0.5
Effects on sexual life	2.142	0.857	0.428
Effects on daily activities	2.375	0.875	0.5

*Note:* Progression of the mean scores for each symptom across all patients. The values refer to symptom assessments conducted before treatment, one month after treatment, and one year after the initiation of therapy. Each symptom is scored on a scale from 0 to 3, with 0 indicating absence, 1 mild, 2 moderate, and 3 severe. An improvement was observed in all symptoms.

Abbreviation: BCS, breast cancer survivors.

### 3.9 Epithelial Thickness

No significant alterations were found in epithelial thickness following treatment (250.4 ± 75.3 μm pre‐treatment vs. 260.7 ± 80.2 μm post‐treatment; *p* = 0.40).

### 3.10 Glycogen Content

At baseline, glycogen was classified as moderate in four patients and absent or minimal in the others; none exhibited severe glycogen levels. After treatment, five patients demonstrated moderate levels and one presented with severe glycogen deposition, while the remaining participants continued to show low or mild glycogen content.

### 3.11 Collagen Fiber Density (Picrosirius Red Staining)

**FIGURE 5:**
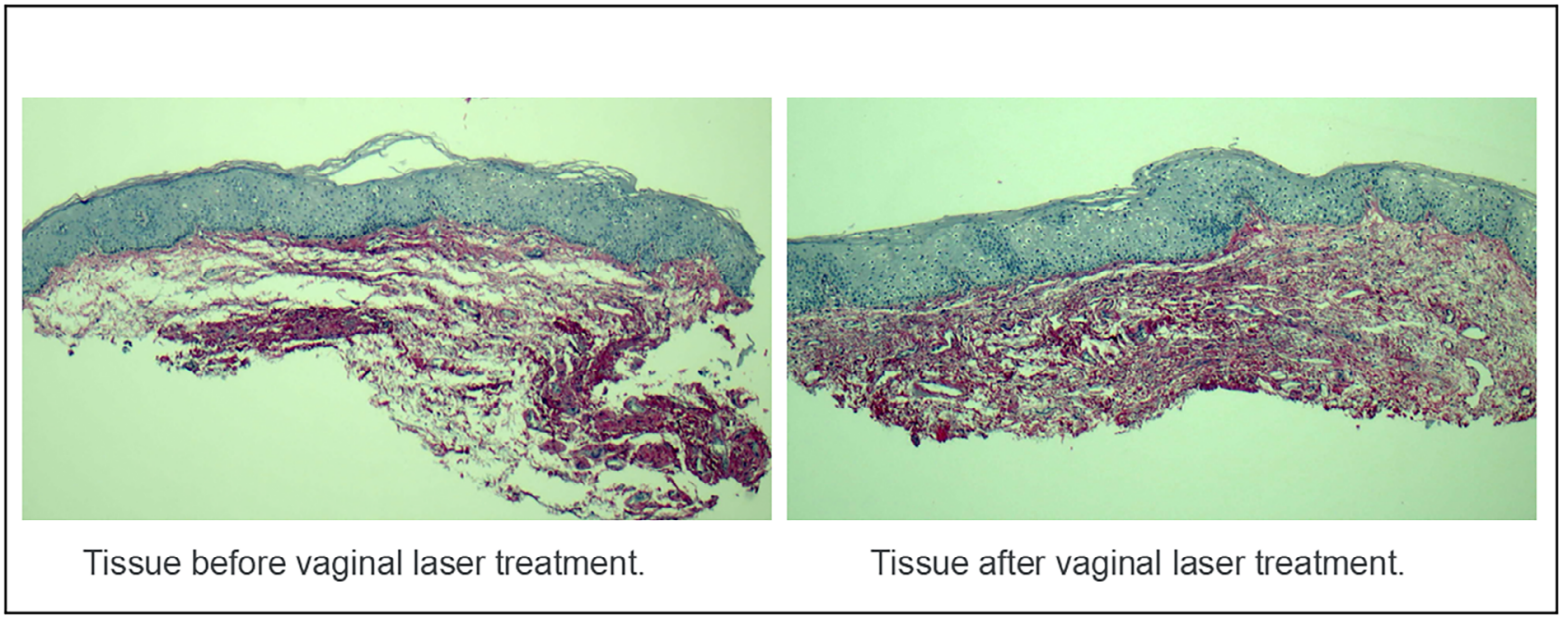
Optical microscopy image of skin biopsy in special Picrosirius stain for collagen: This image displays two fragments of vaginal mucosa—one obtained before and the other after treatment with vaginal Erbium:YAG laser—captured under optical microscopy using special Picrosirius (Sirius Red) staining for collagen. An increase in collagen fiber density is evident in the fragment collected one month post‐treatment.

Although a subjective increase in collagen fiber density was observed via Picrosirius Red staining, quantitative image analysis using Python‐based software did not yield statistically significant changes. The mean collagen‐stained area was 72.3% ± 6.5% before treatment and 74.1% ± 5.8% afterward (*p* = 0.41). Conversely, a statistically significant reduction in the number of epithelial cell layers was observed following the treatment protocol (*p* = 0.0016), suggesting epithelial remodeling and structural reorganization of the vaginal mucosa (mean number of layers: 23.4 ± 3.2 pre‐treatment vs. 18.7 ± 2.9 post‐treatment). Figure 5 illustrates the histological modifications in vaginal biopsies stained with Picrosirius Red, comparing pre‐ and post‐treatment samples from breast cancer survivors presenting with vulvovaginal atrophy.

## 4 Discussion

Studies indicate that the global incidence of breast cancer has increased over the past decades [26]. Epidemiological research data, such as that presented by Bray et al., demonstrate that although incidence is rising, mortality rates have stabilized or even decreased in certain regions—a reflection of therapeutic advances and early detection [27]. Consequently, the population of so‐called breast cancer survivors is growing.

New studies have emerged demonstrating the safety of treating genitourinary syndrome of menopause (GSM) with topical estrogen in breast cancer survivors [28]; however, the safety of this treatment, particularly in patients with tumors that express estrogen receptors, remains controversial, requiring that the decision be individualized by considering the severity of symptoms and the risk of recurrence [29].

Although the present study focused on Er:YAG laser therapy, the fractional CO_2_ laser has also demonstrated promising results in this population [12]. Clinical evidence indicates that CO_2_ laser is a viable and well‐tolerated intervention, capable of improving vaginal symptoms, sexual function, and quality of life without serious adverse events [12]. Furthermore, more recent data support the sustained efficacy of fractional CO_2_ laser, reinforcing its potential as a long‐lasting therapeutic option for women not eligible for hormone‐based treatment [30]. Collectively, these findings underscore the relevance of energy‐based therapies, such as Er:YAG and CO_2_ lasers, as viable alternatives for the management of GSM in breast cancer survivors.

Corroborating other studies that have demonstrated the safety of laser treatment [31, 32, 33, 34] our study concluded without any adverse events. One advantage of laser treatment is maintaining long‐term results—approximately 12 months—without needing continuous application as with topical treatments, which improves treatment adherence [23].

We observed in this study a limitation in the use of the Female Sexual Function Index (FSFI) to evaluate the outcome of laser treatment since other factors can influence the quality of sexual health. However, vaginal health influences sexual health; it does not exclusively determine sexual health. Some patients did not have partners during the study, which reduced the number of patients evaluated by the FSFI.

A study by Gaspar et al. in 2020 [35] reported histological improvement in all ten patients studied. Unlike this study, the improvement reported by Gaspar predominated in the epidermis. A marked increase in epithelial cellularity was reported, with a greater number of layers and greater epithelial thickness, which was accompanied by a significant increase in glycogen load, new papillae, and neoangiogenesis in the lamina propria, with capillaries reaching the epithelium. Despite the findings, the authors acknowledge the difficulty in accurately measuring epidermal thickness, which our researchers also encountered.

Another study, also with a histological study, a study carried out by Fernandes et al. in 2023 [36] reported a dissociation between the symptoms of vaginal atrophy and histological atrophy. In this study, although all patients presented symptoms of moderate to severe atrophy, 90% did not meet the criteria for histological atrophy, demonstrating a dissociation between clinical presentation and histology and indicating that what we call genitourinary atrophy did not necessarily present with histological criteria for atrophy.

The histological changes related to genitourinary syndrome of menopause (GMS) are observed by Salvatore et al. [37] as follows: a change in the proportion of type I collagen fibrils to type III collagen fibrils with a loss of their trabecular arrangement (the collagen fibrils become flattened); a decreased amount of metallurgical fibers; reduced vascularization; and thinning and flattening of the vaginal epithelium, which may superficially transform into a keratinized layer [36]. In our study, all patients presented Vaginal Health Index scores and symptom intensities consistent with moderate to severe atrophy before treatment; however, no histological findings compatible with the reference criteria were found. Although some patients showed significant improvement in collagen fiber density (Figure 4), this pattern was not reproduced in the entire sample, resulting in a lack of statistical significance for these classifications, which can be explained by the small number of patients. Another limitation related to histological evaluation is the difficulty in accurately locating the sample collection site before and after treatment, making it difficult to collect samples from the same site.

## 5 Conclusion

We concluded that the Vaginal Health Index and the Symptom Intensity Questionnaire were the tools that most accurately reflected the clinical reality of the patients in our study. Vaginal Erbium:YAG laser appears to be a safe and effective option for the treatment of genitourinary syndrome of menopause. However, larger‐scale and longer‐term clinical trials are warranted to fully establish their efficacy and safety. Such studies will offer a more comprehensive understanding of this therapeutic approach and validate the present study's findings.

## Ethics Statement

The study was conducted in accordance with the Declaration of Helsinki, and approved by the Ethics Committee of Research Ethics Committee of the Faculty of Medical Sciences of Minas Gerais—FCMMG (protocol code CAAE 75613723.5.0000.5134).

## Conflicts of Interest

Gisele Viana de Oliveira became speaker for other technologies of Vydence after the study was conducted, but received no support regarding this study. The other authors declare there is no conflicts of interest.

## Data Availability

Data available on request from the authors. The data that support the findings of this study are available from the corresponding author upon reasonable request.
